# Impact of federalization for health financing and workforce in Nepal

**DOI:** 10.1186/s41256-023-00304-3

**Published:** 2023-06-08

**Authors:** Meifang Chen, Dinesh Thapa, Rongxiao Ma, Daniel Weissglass, Hao Li, Biraj Karmachaya

**Affiliations:** 1 Division of Social Science, Global Health Research Center, Duke Kunshan University, Kunshan, Jiangsu China; 2 School of Public Health/Global Health Institute, Wuhan University, Wuhan, China; 3 Department of Public Health and Community Program, Dhulikhel Hospital, Kathmandu University Hospital, Kathmandu, Nepal; 4 Division of Arts and Humanities, Global Health Research Center, Duke Kunshan University, Kunshan, China

**Keywords:** Federalization, Decentralization, Financing, Workforce, Nepal, Non-communicable diseases (NCDs), Universal Health Care (UHC), LMICs

## Abstract

The adoption of its 2015 constitution has converted Nepal to a federal government while simultaneously resulted in significant reforms of the health system in Nepal in terms of both structure and commitment. In this commentary, we review evidence ranging from health financing to health workforce development to show that the impact of federalization on Nepal’s health system and its efforts to achieve equitable and affordable universal health care have been mixed. On the one hand, careful efforts of the federal government to support subnational governments during the transition appears to have avoided serious disruption, subnational governments have successfully taken on the financial burden of the health system, and increase subnational control has allowed more flexible adaptation to changing needs than might have otherwise been possible. On the other hand, financing resource and ability disparities across subnational governments contributes to significant disparities in workforce development, and subnational authorities appear to have underestimated significant health issues (e.g. NCDs) in their budgets. We then provide three recommendations to improve the success of the Nepalese system: (1) to assess whether the services covered by health financing and insurance schemes like the National Health Insurance Program adequately address the needs of the rising burden of NCDs in Nepal, (2) to set clear minimum requirements on key metrics for subnational health systems, and (3) to extend grant programs to address resource disparities.

## Background

With the adoption of a new constitution in 2015, Nepal formally transitioned from a unitary to federal government, placing greater authority in subnational governments (SNGs). The constitution also places asserts that health is a human right, and commits to providing equitable and affordable universal health care (UHC) through making basic health services free-of-charge, while ensuring that other services available at an affordable cost. Together, these aspects of the new Nepalese constitution amount to significant changes to the structure and commitments of the Nepalese health care system. This commentary assesses the impact of federalization on health financing and workforce—two critical determinants of the success of the Nepalese health care system. It then provides recommendations to help Nepal better provide for the health needs of the Nepalese people.

## Health system in Nepal

**Fig. 1:**
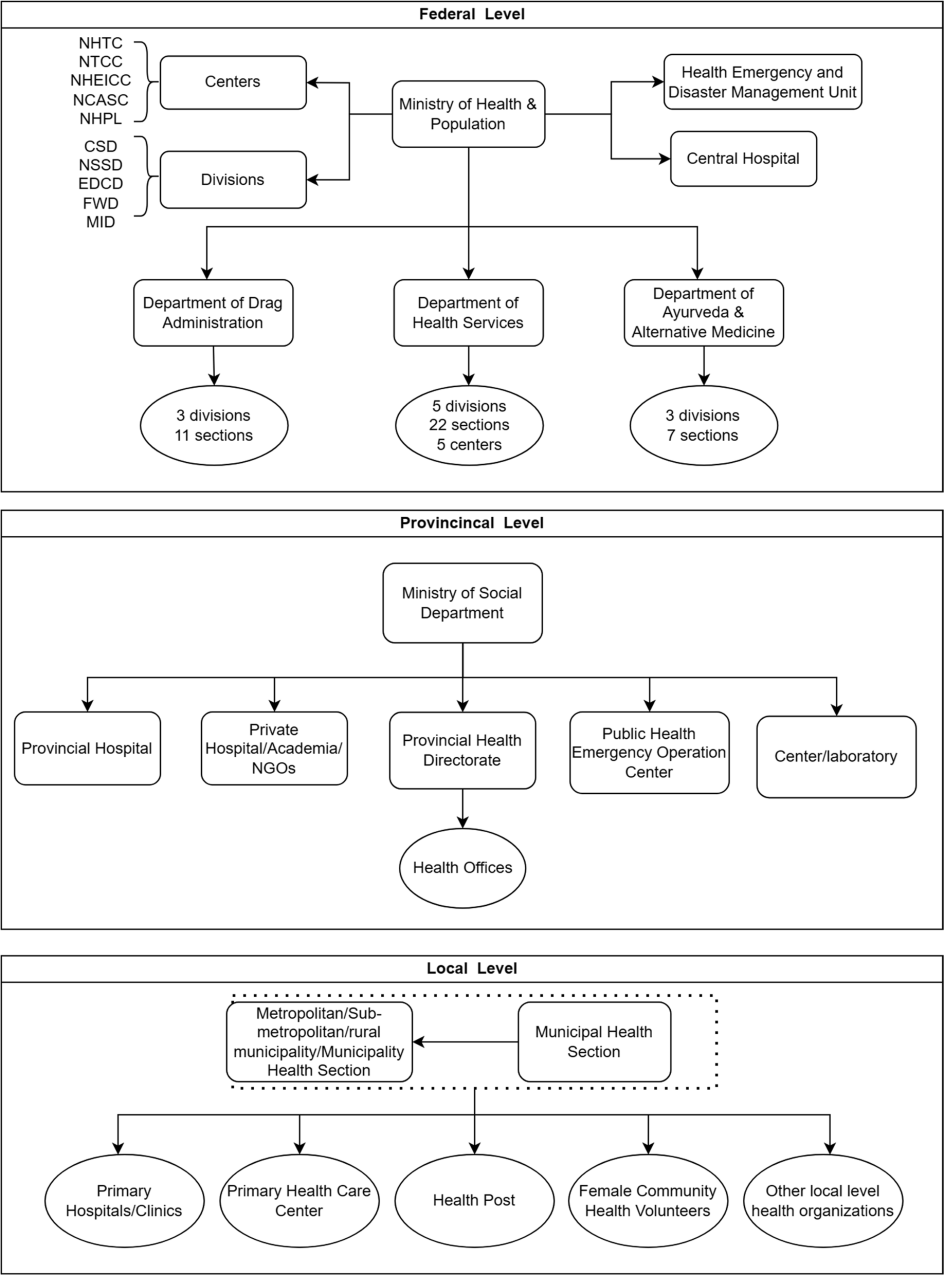
Organizational structure of the reformed health system of Nepal. Source: The authors (Meifang Chen and Rongxiao Ma), prepared using information from [2, 3] (Note:‘NHTC’ = National Health Training Centre; ‘NTC’ = National Tuberculosis Control Centre; 'NHEICC' = National Health Education, Information and Communication Centre; 'NCASC' = National Centre for AIDS and STD Control; ‘NHPL’ = National Public Health Laboratory; ‘CSD’ = Curative Service Division; ‘NSSD’ = Nursing and Social Security Division; ‘EDCD’ = Epidemiology and Diseases Control Division; ‘FWD’ = Family Welfare Division; ‘MID’ = Management Division)

The modern Nepalese health care system, dating to the 1950’s, has gone through several periods of development. From the 1960’s to 1990’s, development was concentrated in the expansion of health facilities and the implementation of vertical health interventions. The 1990’s saw a shift towards privatization alongside a national health policy designed to increase access to health care to establish modern health care facilities across Nepal, collectively creating conditions for a rapid expansion of the primary care system and increasing influence of private health care facilities [1]. With the 2015 constitution, the Nepalese health care system has been given new expectations—to promote UHC—and a new structure which places Nepal’s 7221 public health facilities under the jurisdiction of any one of the federal government, 7 provincial governments, or 753 local governments (Fig. 1).

Reports from health care workers in Nepal suggest both benefits and costs of the federalization process in efforts to provide UHC and address significant health issues. These reports suggest that federalization has made the health care system flexible and responsive, able to adjust to changing needs and reflect the value of the community. Investments could be made, policies adapted, and workforce recruited more easily by local governments without dependence on oversight by the central government. However, they also suggest a system which is divided and inequitable. Local governments lacking resources end up unable to provide care for their citizens, conflicting policies across levels creates uncertainty, and the commitment and qualification of local leadership to promote health varies widely [4]. These complicated and conflicted reports suggest that careful attention should be directed to ensuring that the Nepalese health care system is able to maximize the benefits of federalism while avoiding its risks. This paper explores these issues with respect to health financing and workforce development, two major determinants of the success of UHC.

## Health financing post-federalization

In line with its commitment to accelerate UHC, and in an effort to prevent disruption during the period of federalization, the federal government has significantly increased the health budget, with the health spending as % of GDP increasing from 1.5% in 2016/17 to 2.4% in 2020/21, and has allocated budget through equalization, conditional, special and complementary grants to help new SNGs establish effective health systems [5, 6]. The result has been a largely successful transition of the Nepalese health care system to a federal financing model, with SNGs having successfully taken on much of the burden of financing the health care system. The proportion of health care expenditures taken on by SNGs steadily increasing since federalization, and the role of sources of funding internal to SNGs (e.g., taxes gathered by provincial governments) has grown rapidly from nearly 0% immediately after federalization (2017/2018) to nearly 64% in 2021/2022 [5]. This has been coupled with general efforts on the part of the federal government to support the affordability of health care through insurance schemes like the National Health Insurance Program (NHIP), which is meant to prevent health expenses which impoverish citizens—a critical step towards UHC. Collectively, these represent considerable resources put forward by the federal government to support the transition to federalism and the achievement of UHC.

The patterns of SNG health expenditures, however, exhibit a failure to match funding to needs, as is especially apparent in the case of NCD management. The NCD burden of Nepal is rapidly increasing, with the proportion of all deaths in Nepal attributable to NCDs in 2019 (71.1%) is more than double that of 1990 (31.3%), recent local expenditures have not reflected the urgent risk of NCDs [7]. In fiscal year 2021/2022, provincial governments allocated less than 4%, and local governments less than 1%, of their expenditures to NCD management. This has placed the burden of financing NCD treatment on private citizens. For instance, nearly all (97.4%) patients with diabetes in Kathmandu paid out of pocket for diabetes treatments, while patients with diabetes spent a monthly average of NPR 7312.17 at public hospitals or NPR 10,125.31 at private hospitals. As the per capita income of Nepal was NPR 103,335 in the same fiscal year this is a considerable expense—one that would drive many to poverty, indicating a failure of the NHIP and local government in the case of diabetes [8, 9].

Likewise, variations in SNG health expenditure raises concerns of inequity. Significant variation in health care spending across provinces, in terms of both per capita spending (NPR 384 to NPR 3,338) and proportional spending (0.3% to 2.9% of GDP) [5]. These large ranges—with maxima nearly 8.7 (per capita spending) and 9.7 times (proportional spending) their minima—support reports from health workers that there are disparities in the real resources dedicated to health care across provinces, as well as the relative priority given to funding health care systems [4].

## Impact of federalization on health workforce

Federalization has also had significant impacts on health workforce development in Nepal, placing workforce decision making largely under the control of SNGs. This increased control has been cited favorably by public health officers in Nepal as allowing SNGs to rapidly and flexibly adapt their recruitment and training programs to match their workforce needs. This suggests that federalism has brought significant advantages in allowing the health system to fit workforce practices to needs [4].

This flexibility, however, has also been cited as a cause of uneven and inequitable health workforce development across regions, both in quality and quantity. While some regions have the necessary resources and leadership to recruit and train adequate workforce, and there is considerable variation in compensation across SNGs [4]. We can see a suggestion of this effect in the concentration of health workforce development in urban centers (e.g., Kathmandu), which has made access to health care difficult for rural populations [10]. In concert with wide variations in per capita health care expenditure across SNGs, this workforce variation could result in serious disparities in health care access in Nepal.

More broadly, SNGs have failed to adequately support workforce development. The share of the health expenditures for wages and salaries has decreased during federalization from 24.3% in 2017/18 to 13.8% in 2021/22, a decrease at least partly attributable to salary being placed under the direction of SNGs. Likewise, federal funding was still the primary source for efforts to improve staff availability (nearly 100% federal) and training (61%/39%/0%, federal/provincial/local) in fiscal year 2021/2022 [5]. These failures contribute to serious workforce shortages, where key roles go unfilled and qualified health care workers can be hard to find [4].

## Conclusions

Nepal has had mixed success in achieving equitable and affordable UHC post-federalization. Success in financing have been a relatively smooth transition to a federal system, where support from the federal government has allowed SNGs to successfully take over large portions of health care funding in a gradual way, and the development of insurance policies to maintain affordability of care. In workforce development, federalization has enabled flexible recruitment and training. However, federalization has raised considerable concerns about growing disparities and mismatched priorities in both financing and workforce development.

In light of these concerns, three policies may help Nepal capture the greatest benefit from its restructured health care system while minimizing its costs. First, the Nepalese government should regularly review the kinds of care considered sufficiently basic to be provided at no-cost to end users, ensuring that this category captures the most efficient tools for improving health outcomes given Nepal’s current needs. In the face of a rapidly increasing NCD burden, it is likely that this will require giving a correspondingly increasing amount of attention to NCD prevention and treatment. Second, the Nepalese federal government should create minimum requirements for SNGs (e.g., an appropriate provider/patient ratio, availability of key resources, etc.), allowing it to direct SNGs to develop health care systems in line with national priorities while still allowing flexibility with respect to how goals beyond those minima are prioritized. Third, the federal government should plan to maintain grant programs for low-income regions to ensure that they are able to equitably care for their citizens, aiming to counter emerging trends in inequitable funding and workforce development.

## Data Availability

Not applicable.

## Abbreviations

CSD: Curative Service Division
DALYs: Disability-adjusted life years
EDCD: Epidemiology and Diseases Control Division
EPF: Employee provident fund
FWD: Family welfare division
FY: Fiscal year
LMICs: Low- and middle-income countries
MID: Management division
NCASC: National Centre for AIDS and STD Control
NCDs: Non-communicable diseases
NHEICC: National Health Education, Information and Communication Centre
NHIP: National health insurance program
NHPL: National Public Health Laboratory
NHSP: Nepal Health Sector Plan
NHTC: National Health Training Centre
NPR: Nepalese Rupees
NSSD: Nursing and Social Security Division
NTC: National Tuberculosis Control Centre
PHC: Primary health care
SSF: Social Security Fund
UHC: Universal health care
USD: United States dollar
